# Supplementary material for: Short-term dietary fiber interventions produce consistent gut microbiome responses across studies
Source: mSystems. 2024 May 14;9(6):e00133-24. doi: 10.1128/msystems.00133-24 (PMC11237734; doi:10.1128/msystems.00133-24)
Supplement: Table S1 — Betadisper results. [file msystems.00133-24-s0001.docx]

**Supplemental Table 1.** Betadisper results to test beta dispersion between the before vs after fiber intervention groups.

| **Study Name** | **Betadisper P-value centroids** | **Significant?** |
| --- | --- | --- |
| Baxter_2019_V4_himaize (RMS) | 0.82 | No |
| Baxter_2019_V4_inulin | 0.594 | No |
| Baxter_2019_V4_potato (RPS) | 0.366 | No |
| Dahl_2016_V1V2_potato-RS4A (RPS) | 0.719 | No |
| Dahl_2016_V1V2_potato-RS4B (RPS) | 0.465 | No |
| Dahl_2016_V1V2_potato-RS4C (RPS) | 0.577 | No |
| Deehan_2020_V5V6_maize-RS4 | 0.038 | Yes |
| Deehan_2020_V5V6_potato-RS4 | 0.806 | No |
| Deehan_2020_V5V6_tapioca-RS4 | 0.1 | No |
| Healey_2018_V3V4_inulin-FOS | 0.467 | No |
| Hooda_2012_V4V6_corn | 0.518 | No |
| Hooda_2012_V4V6_polydextrose | 0.214 | No |
| Kovatcheva_2015_V1V2_kbb | 0.232 | No |
| Liu_2017_V4_FOS | 0.532 | No |
| Liu_2017_V4_GOS | 0.883 | No |
| Morales_2016_V3V4_oligofructose | 0.338 | No |
| Rasmussen_2017_V1V3_psyllium | 0.166 | No |
| Rasmussen_2017_V1V3_SM12 | 0.751 | No |
| Tap_2015_V3V4 | 0.879 | No |
| Vandeputte_2017_V4_inulin | 0.712 | No |
| Venkataraman_2016_V4_potato (RPS) | 0.405 | No |
